# Rapid Prototyping Flexible Aortic Models Aids Sizing of Valve Leaflets and Planning the Ozaki Repair

**DOI:** 10.1016/j.jaccas.2020.04.054

**Published:** 2020-07-15

**Authors:** Andrew I.U. Shearn, Maria Victoria Ordoñez, Filippo Rapetto, Massimo Caputo, Giovanni Biglino

**Affiliations:** a Bristol Heart Institute, University Hospitals Bristol, NHS Foundation Trust, Bristol, United Kingdom; b Bristol Medical School, Faculty of Health Sciences, University of Bristol, Bristol, United Kingdom; c CRIC Bristol, University of Bristol, Bristol, United Kingdom; d National Heart and Lung Institute, Imperial College London, London, United Kingdom

**Keywords:** 3D printing, aortic coarctation, bicuspid aortic valve, computed tomography, Ozaki repair, rapid prototyping, surgical planning, valve repair, 3D, 3 dimensional, AR, aortic regurgitation, BAV, bicuspid aortic valve, CT, computed tomography, V_max_, maximum velocity

## Abstract

Two patients with bicuspid aortic valve were selected for aortic valve repair using the Ozaki procedure. Patient-specific models of their aortic roots were generated based on computed tomography data and were 3-dimensional printed using a flexible resin. The models allowed sizing of the valve leaflets and practicing of leaflet suturing. (**Level of Difficulty: Advanced.**)

## Graphical abstract

**Figure:**
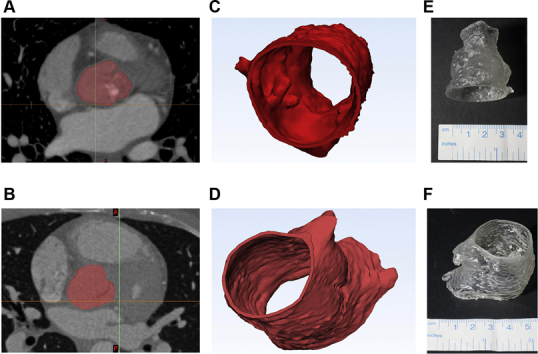

Two patients with a diagnosis of bicuspid aortic valve (BAV) were selected for aortic valve repair using the Ozaki procedure. Patient #1 was a 65-year-old woman with functional BAV, asymptomatic moderate-to-severe aortic stenosis, and a small anterior communicating artery aneurysm who was followed up at the University Hospitals Bristol congenital heart disease clinic. Detriment of her functional capacity was observed on cardiopulmonary stress testing. Echocardiographic examination showed severe aortic stenosis, with an aortic valve area of 0.5 cm^2^, maximum velocity (V_max_) of 4.5 m/s, mean gradient of 45 mm Hg, and normal systolic left ventricular function. The decision was taken to intervene surgically.

**Learning Objectives**

• To understand the role of 3D printing models in surgical planning for aortic valve repair.
• To appreciate decision-making aspects around aortic valve repair with the Ozaki technique in cases of BAV with different annulus sizes.

Patient #2 was a 34-year-old man with congenital BAV and coarctation of the aorta repaired at the age of 4 years with end-to-end anastomosis and residual hypertension. He presented with symptoms of breathlessness and progression of aortic regurgitation (AR) due to leaflet prolapse. Echocardiographic examination confirmed severe AR, with normal ejection fraction and a dilated left ventricle. Surgical treatment was planned. Detailed patient characteristics are summarized in Table 1.

**Table 1:** Patient Characteristics

	Patient #1	Patient #2
Demographic data		
Age at operation, yrs	65	34
Sex	Female	Male
Anatomy	Functional BAV	BAV (LCC-RCC), coarctation of the aorta repair at age 4 yrs
Treatment	—	—
Surgical indication	Detriment of functional capacity	Progression of AR
ECG	SR, 75 beats/min	SR, 56 beats/min
Weight, kg	68	91
Height, cm	176	184
BSA, cm^2^	2.2	2.6
Hypertension	No	Yes
Echocardiogram data		
Aorta V_max_, m/s	4.5	1.9
Peak gradient, mm Hg	75	—
Mean gradient	45	—
Aortic regurgitation	Mild	Severe
EF, %	55	65
lS′-wave, cm/s	10	11
sS′-wave, cm/s	8	9
E/A	1.0	1.6
E/E′	7	7.9
LVEDD, mm	42	68
LVESD, mm	26	55
CT data		
Aortic annulus, mm	24	33 × 34
SV, mm	31	40 × 34
Ascending aorta, mm	38 × 38	30 × 32
Descending aorta, mm	20	25
CT acquisition		
Columns, n	512	512
Slice thickness, mm	0.60	0.50
Pixel spacing, mm	0.39	0.34
Cycle time acquisition	End systole	Diastole

AR = aortic regurgitation; BAV = bicuspid aortic valve; BSA = body surface area; CT = computed tomography; ECG = electrocardiography; EF = ejection fraction; LCC = left coronary cusp; LVEDD = left end-diastolic diameter; LVESD = left end-systolic diameter; RCC = right coronary cusp; SR = sinus rhythm; SV = sinus of Valsalva; V_max_ = maximum velocity.

At the multidisciplinary meeting, the decision to carry out aortic leaflet reconstruction using glutaraldehyde-treated autologous pericardium—the Ozaki procedure (1,2)—for both patients was based on the advantages of avoiding anticoagulation (necessary for a mechanical valve prosthesis) and the potentially better longevity of autologous material compared with a biological valve prosthesis. A Ross procedure was also discussed but discounted. Both patients fully accepted the concept of autologous pericardial reconstruction of the aortic leaflets and the available evidence in the literature (2).

As a potential aid to leaflet sizing, patient-specific models of the aortic root for each case were generated based on the patients’ computed tomography (CT) data (Figures 1A and 1B, Videos 1 and 2). Their CT data sets were imported into and processed with commercial software (Mimics, Materialise, Leuven, Belgium) for 3-dimensional (3D) reconstruction (3). A 3D volume of the aortic root was generated (Figures 1C and 1D) and exported to a 3D printer. Aortic root models were printed in house (Form2, Formlabs, Somerville, Massachusetts) by using a soft and resilient compliant compound (Elastic Resin, Formlabs; mechanical properties as per manufacturer’s data sheet: elongation at break: 160%; tensile strength: 3.2 MPa; tear strength: 19.1 kN/m), with a wall thickness of 1 mm. Once manufactured, the models were provided to the surgeon for leaflet sizing and suturing.

**Figure 1:**
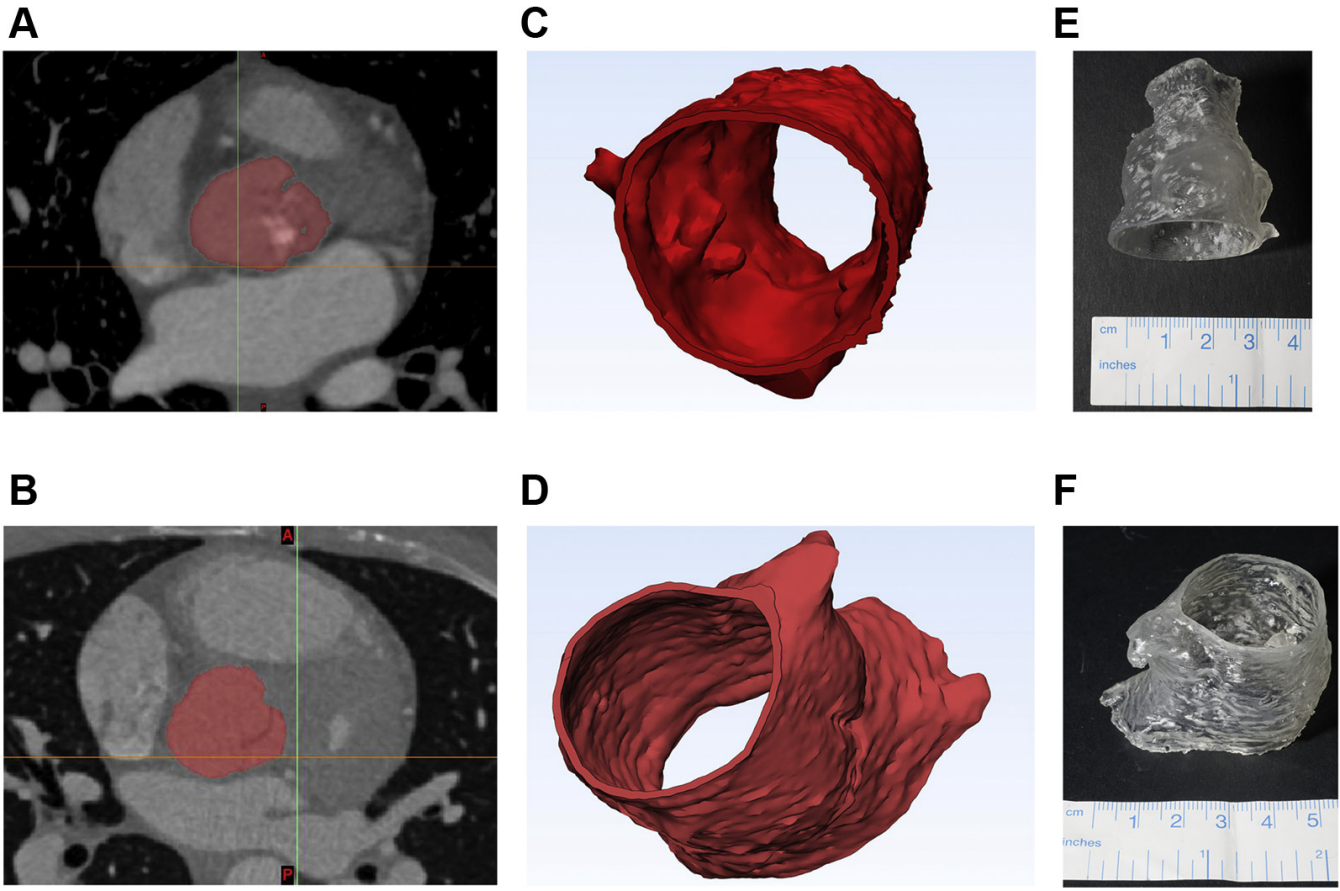
Patient-Specific Models of the Aortic Roots Were Derived From Clinically Indicated Computed Tomography Datasets **(A, B)** Segmentation was carried out by using Materialise (Leuven, Belgium) Mimics software to select the area of interest **(highlighted in red)**—in this case, the aortic root. **(C, D)** Materialize 3-matic was used to reconstruct the aortic roots in 3 dimensions (3D) and produce an stereolithography file suitable for importing into the 3D printer software. **(E, F)** The aortic roots were then printed in a flexible resin. Images for Patients #1 and #2 displayed are in the **top and bottom rows**, respectively.

**Online Video 1:** Aortic root model reconstructed from CT data.

**Online Video 2:** Aortic root model reconstructed from CT data.

Models were successfully produced for both types of aortic valve disease (Figures 1E and 1F), demonstrating the feasibility of the workflow for pre-sizing aortic valve leaflets in 2 patients with BAV with different underlying causes of valve dysfunction. Models took approximately 1 hour to reconstruct and 6 h to print each. Once provided to the surgeon, the sizing process was successfully performed in the lab (Figure 2A, Table 1, Video 3), and leaflets were cut from GoreTex to practice suturing. Leaflet suturing was also demonstrated to be feasible (Figure 2B), and feedback from the surgeon was extremely positive, highlighting the qualities of the material and the advantage, for prospective cases, of pre-sizing the aortic valve leaflets.

**Figure 2:**
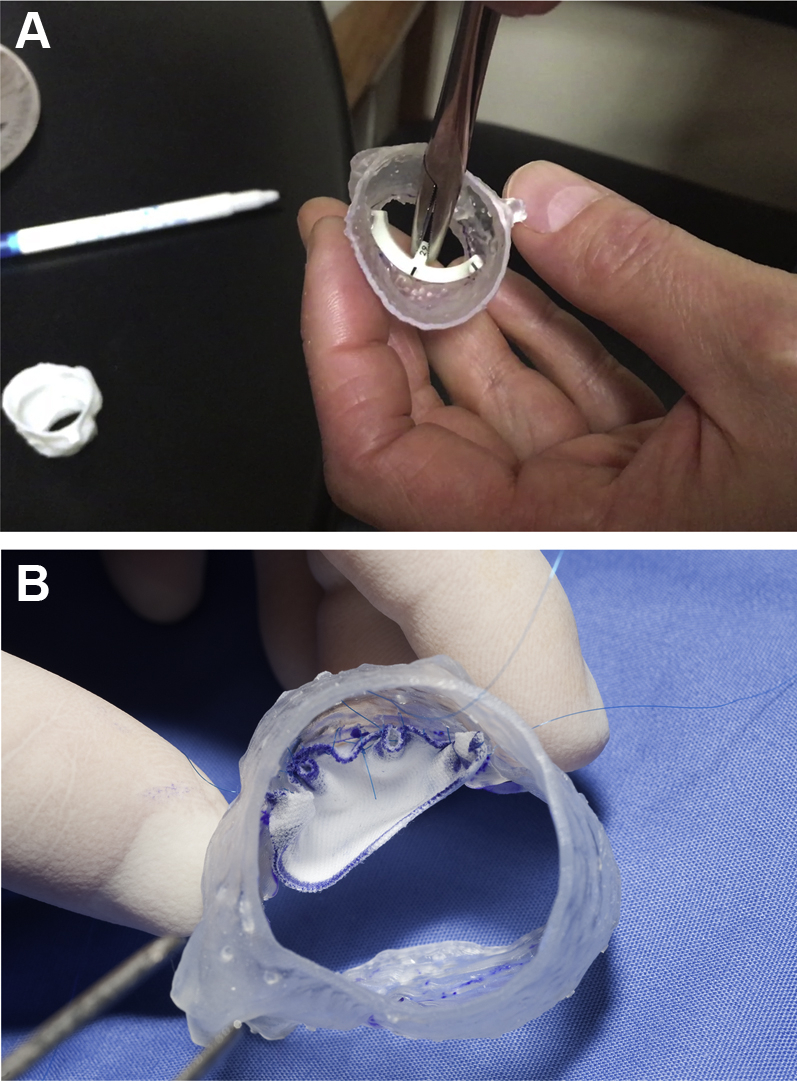
Examples of Using the Aortic Root Model Examples of using the model by **(A)** sizing using the Ozaki sizers and **(B)** practicing leaflet suturing.

**Online Video 3:** Sizing of the aortic sinuses on 3D printed model.

With regard to surgical results, Patient #1 did not present any complications after surgery and was discharged after 5 days. The echocardiogram at 4 weeks post-surgery showed no AR, aortic V_max_ of 2.2 m/s, a substantially reduced mean gradient of 10 mm Hg, and aortic valve area of 1.5 cm^2^. Patient 2 was also discharged 5 days after surgery without complications. An echocardiogram at 4 weeks post-surgery showed no AR, an aortic V_max_ of 1.8 m/s, and a reduction in LV dimensions (Table 2).

**Table 2:** Post-Operative Results

	Patient #1	Patient #2
Leaflet sizing		
Leaflet sizes, intraoperative, mm		
RCC	27	27
LCC	25	27
NCC	27	31
Leaflet sizes, model, mm		
RCC	29	33
LCC	27	31
NCC	33	35
Echocardiogram data		
E/A	1.5	1.6
E/E′	7.5	7.9
LVEDD, mm	38	44
LVESD, mm	27	31

NCC = noncoronary cusp; other abbreviations as in Table 1.

3D printing technology is increasingly recognized as a valuable tool for surgical planning, providing an insight into complex intracardiac structures with accurate sizing and providing the surgeon with the ability to visualize the heart before the operation (3). These cases highlight that the Ozaki procedure is a novel scenario in which the possibility of replicating patient-specific anatomies by means of 3D printing technology can be of benefit. The Ozaki procedure is a cutting-edge approach to aortic valve reconstruction with very favorable initial results (4). It is a technique that uses stentless aortic valve replacement and uses autologous pericardium for the reconstruction of the valve leaflets (5). Sizing of the leaflets currently takes place in the surgical theater but, as demonstrated here, could instead be planned ahead, thereby saving time in the operating theater. We note that the pre-sized leaflets were slightly bigger than those ultimately implanted in the patients during surgery, allowing for the surgeon to trim the leaflets once implanted if deemed appropriate/necessary. Furthermore, in reoperation scenarios, when autologous pericardium cannot be used, a pre-operative 3D printing–based aortic valve leaflet using bovine or tissue engineered material could, potentially, help the planning and the execution of the procedure in a patient-specific manner.

The precision of the 3D printing manufacturing process (6,7) enables accurate anatomic replicas to be produced to facilitate patient counseling, offer training opportunities, and inform the clinical decision-making process. In the case of the Ozaki procedure, when suitable imaging data (CT or cardiovascular magnetic resonance) are available, patient-specific aortic root models can be printed in a short time frame, allowing the surgeon to size and prepare the aortic valve leaflets before the actual surgery.

These 2 cases of aortic valve disease with different annulus sizes were successfully reproduced using 3D models, and the short-term outcomes following the Ozaki repair in these patients were excellent, based on an improvement in functional capacity and echocardiography.

## Take-Home Message

In light of the experience presented here, further research into 3D printing patient-specific aortic models for surgical planning in valve repair/replacement is certainly warranted, including exploring novel materials (e.g., silicone) compatible with the technology and testing these in a systematic manner. We will also look to expand this study to a larger case series of patients, potentially including longer follow-up.
